# Fumonisin B1: A Tool for Exploring the Multiple Functions of Sphingolipids in Plants

**DOI:** 10.3389/fpls.2020.600458

**Published:** 2020-10-27

**Authors:** Hong-Yun Zeng, Chun-Yu Li, Nan Yao

**Affiliations:** ^1^State Key Laboratory of Biocontrol and Guangdong Provincial Key Laboratory of Plant Resource, School of Life Sciences, Sun Yat-sen University, Guangzhou, China; ^2^Institution of Fruit Tree Research, Guangdong Academy of Agricultural Sciences, Guangzhou, China

**Keywords:** fumonisin B1, sphingolipid, long chain bases, ceramides, cell death, plant growth

## Abstract

Fumonisin toxins are produced by *Fusarium* fungal pathogens. Fumonisins are structural analogs of sphingosine and potent inhibitors of ceramide synthases (CerSs); they disrupt sphingolipid metabolism and cause disease in plants and animals. Over the past three decades, researchers have used fumonisin B1 (FB1), the most common fumonisin, as a probe to investigate sphingolipid metabolism in yeast and animals. Although the physiological effects of FB1 in plants have yet to be investigated in detail, forward and reverse genetic approaches have revealed many genes involved in these processes. In this review, we discuss the intricate network of signaling pathways affected by FB1, including changes in sphingolipid metabolism and the effects of these changes, with a focus on our current understanding of the multiple effects of FB1 on plant cell death and plant growth. We analyze the major findings that highlight the connections between sphingolipid metabolism and FB1-induced signaling, and we point out where additional research is needed to fill the gaps in our understanding of FB1-induced signaling pathways in plants.

## Introduction

Fumonisins are produced by several species of *Fusarium* molds such as *Fusarium verticillioides* and *Fusarium moniliforme*, which infect many cereal crops such as maize (*Zea mays*), wheat (*Triticum aestivum*), and barley (*Hordeum vulgare*) (Rheeder et al., 2002). Fumonisins are required for the development of foliar disease symptoms in infected maize seedlings (Glenn et al., 2008). In the field, fumonisins are synthesized *in planta* by fungi and can be taken up by roots and disseminated inside seedlings, possibly affecting the fungus–host interaction prior to the first contact between the pathogen and seedling (Arias et al., 2016). Contaminated corn and corn-based products are frequently ingested in food or feed, causing diseases such as equine leukoencephalomalacia, porcine pulmonary edema, and possibly kidney and liver cancer (as revealed in rats in the laboratory) (Riley and Merrill, 2019).

In 1988, fumonisin B1 (FB1) and FB2 were isolated from *F. verticillioides* (*F. moniliforme*) MRC 826 cultures as novel mycotoxins with cancer-promoting activity in rat livers (Gelderblom et al., 1988). FB1 and FB2 are diesters of propane-1,2,3-tricarboxylic acid with similar long-chain aminopolyol backbones. These compounds are structurally similar to the sphingoid bases sphinganine and sphingosine, with tricarboxylic acid groups added at the C14 and C15 positions (Supplementary Figure S1). However, the chain lengths of their backbones differ, with sphingosine mostly containing 18 carbons and FB1 containing 20 carbons (Supplementary Figure S1). Three years after the chemical structures of fumonisins were elucidated, these carcinogenic mycotoxins were found to disrupt the pathway for *de novo* sphingolipid biosynthesis (Wang et al., 1991). Accumulating evidence has confirmed that the wide spectrum of animal and plant diseases caused by fumonisins are due to changes in the levels of multiple bioactive lipids and related biological processes. Although the mode of action of these toxins has been studied in detail at the cellular level in animals, their role in plant diseases has not been thoroughly elucidated.

**FIGURE 1:**
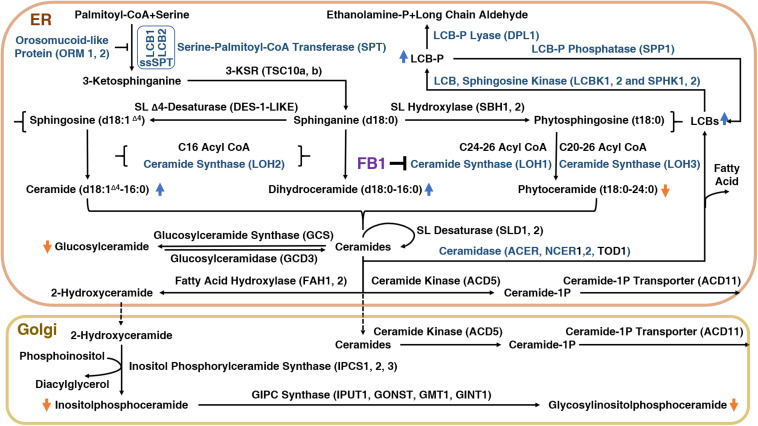
The sphingolipid biosynthetic pathway in plants and the inhibition of this pathway by FB1.

To explore how FB1 affects plants, it is important to understand the plant sphingolipid metabolism pathway (Figure 1). There are five major classes of plant sphingolipids: long-chain bases (LCBs), ceramides (Cers), hydroxyceramides (hCers), glucosylceramides (GluCers), and more complex glycosylated sphingolipids known as glycosyl inositol phosphoryl ceramides (GIPCs). The first four groups are produced in the endoplasmic reticulum (ER), whereas GIPC biosynthesis is initiated in the ER and completed in the Golgi apparatus (Figure 1). The serine palmitoyl-CoA transferase (SPT) protein complex catalyzes *de novo* biosynthesis of LCBs resulting from the condensation of serine and palmitoyl-CoA moieties (Kimberlin et al., 2013). The SPT product is subsequently reduced to form sphinganine, the precursor of the eight other LCBs found in plants.

Ceramide synthases (CerSs) are encoded by a multigenic family named after the yeast protein Lag One Homolog (LOH) and are responsible for the formation of the amide bond that links long-chain (<C20) fatty acids (LCFs) or very-long-chain (≥C20) fatty acids (VLCFAs) to LCB, leading to Cer formation (Markham et al., 2011). Cers can then be phosphorylated to form ceramide 1-phosphate (ceramide-1P) (Liang et al., 2003), or α-hydroxylated at the fatty acid moiety to form hCers (Konig et al., 2012). Cers can also be used as backbones for the production of GluCer and GIPC by the addition of a glucose molecule or an inositolphosphoryl group, followed by one glycosylation step for GluCer or several glycosylations for GIPC (Markham et al., 2013).

Sphingolipid compounds have a multitude of functions. In addition to functioning as structural components of the plasma membrane and other endomembrane systems, sphingolipids serve as bioactive molecules and second messengers for an array of cellular signaling activities in plant cells, such development, biotic and abiotic stress responses, and programmed cell death (PCD). However, compared with animals, much less is known about sphingolipid metabolism and signaling in plants. For instance, the receptors, targets, and mediators involved in these processes are almost totally unclear (Ali et al., 2018), limiting our understanding of the FB1 signal transduction pathway in plants.

Schematic representation of the sphingolipid biosynthetic pathway in plants. 3-KSR, 3-ketosphinganine reductase; ACD11, accelerated cell death 11, Cer-1P transporter; ACD5, accelerated cell death 5, ceramide kinase; ACER, alkaline ceramidase; CoA, coenzyme A; d18:1^Δ4^, sphingosine; d18:0, sphinganine; DPL1, dihydrosphingosine phosphate lyase; FAH1/2, fatty acid hydroxylase; GCS, glucosylceramide synthase; GINT1, glucosamine inositolphosphorylceramide transferase 1; GMT1, GIPC mannosyl-transferase 1; GONST1, Golgi localized nucleotide sugar transporter 1; IPC, inositol phosphorylceramide; IPUT1, inositol phosphorylceramide glucuronosyltransferase 1; LCB, long-chain-base; LCB1/2, subunit of serine palmitoyltransferase 1/2; LCBK1/2, long-chain base kinase 1/2; LCB-P, long-chain-base phosphate; LOH1/2/3, LAG1 Homolog 1/2/3, ceramide synthase; NCER, neutral ceramidase; ORM1/2, orosomucoid-like protein 1/2; SBH1/2, sphingoid base hydroxylase 1/2; DPL1, dihydrosphingosine phosphate lyase; SL, sphingolipid; SPHK1/2, sphingosine kinase 1/2; SPP1, sphingoid phosphate phosphatase; SPT, serine palmitoyl transferase; ssSPT, small subunit of serine palmitoyl transferase; t18:0, phytosphingosine; TOD1, turgor regulation defect 1.

## FB1 Disrupts Sphingolipid Homeostasis in Plants

### Inhibition of Ceramide Synthase Activity

In mammals, FB1 inhibits the activity of the six known CerSs, which differ in terms of tissue distribution and fatty acyl-CoA specificity (Cingolani et al., 2016). Tomato (*Solanum lycopersicum*) Alternaria stem canker resistance-1 (Asc-1) was the first CerS identified in plants (Brandwagt et al., 2000; Spassieva et al., 2002). A tBLASTn search of the complete *Arabidopsis thaliana* genome using the sequence of the CerS gene longevity assurance gene 1 (*LAG1*) from yeast and *Asc-1* as queries identified three Asc-1/LAG1 Homologs, *LOH1–3* (Markham et al., 2011). Phylogenetic analysis revealed that LOH1 and LOH3 are closely related and belong to a cluster with most other plant LAG1 homologs, but LOH2 appears to be evolutionarily distinct from LOH1 and LOH3 (Markham et al., 2011; Ternes et al., 2011).

The varied chemical compositions of Cers in Arabidopsis are determined by the specificity of three different isoforms of CerS for a range of LCBs and acyl-CoA substrates. LOH1 and LOH3 prefer very-long chain acyl-CoA and trihydroxy LCB as substrates, but LOH2 prefers palmitoyl-CoA and dihydroxy LCB as substrates (Markham et al., 2011; Ternes et al., 2011; Luttgeharm et al., 2015, 2016). The differences between the two groups of CerSs are reflected by the phylogenetic separation of the *LOHs*, which preceded the separation of mosses and vascular plants (Ternes et al., 2011). Consistent with their redundant biochemical functions, LOH1 and LOH3 share high sequence similarity and a more recent evolutionary origin (Ternes et al., 2011). Nevertheless, when the physiological functions of these CerSs were examined by mutant analysis, only *loh1* plants showed spontaneous cell death, perhaps due to the higher expression level of *LOH1* and the stronger contribution of LOH1 to Cer biosynthesis compared with LOH3 (Ternes et al., 2011). *In vitro* ceramide synthase assays showed that LOH1 was the most sensitive to inhibition by FB1 (Luttgeharm et al., 2016). Furthermore, overexpressing *LOH1* had no effect on FB1 resistance in Arabidopsis, whereas overexpressing *LOH2* or *LOH3* resulted in increased plant resistance to FB1 treatment, confirming the notion that LOH1 confers FB1 sensitivity to plants (Luttgeharm et al., 2015). In line with these observations, *LOH2*- and *LOH3*-overexpressing plants had reduced levels of free LCBs and LCB-Ps in response to FB1 treatment (Luttgeharm et al., 2015).

It remains unknown why FB1 preferentially inhibits LOH1 rather than LOH3, which share similar properties, but have slightly different substrate binding pockets. Supporting the hypothesis that the difference in substrate binding pockets affects their inhibition by FB1, LOH1 prefers FB1-like saturated trihydroxy LCBs, but LOH3 prefers unsaturated trihydroxy LCB (Luttgeharm et al., 2016). It is unclear if LOH1 and LOH3 in other plant species show parallel differences in substrate preference. Notably, in tomato, the presence of Asc-1 relieves blocked sphingolipid synthesis caused by AAL toxin (a tomato-specific toxin produced by the fungus *Alternaria alternata* f.sp. *lycopersici*) (Supplementary Figure S1), which would otherwise lead to PCD (Spassieva et al., 2002), pointing to the presence of at least one more ceramide synthesis enzyme besides Asc-1. Notably, maize (*Z. mays*) likely contains at least two ceramide synthase isoforms with different substrate specificities, which may result in susceptibility/resistance to *F. verticillioides* (Arias et al., 2016).

### FB1 Treatment Increases LCB and C16 Sphingolipid Levels and Reduces VLC Sphingolipid Levels

When applied to plants, FB1 triggers the accumulation of LCBs and LCB-Ps (Shi et al., 2007; Tsegaye et al., 2007; Saucedo-García et al., 2011b; Yanagawa et al., 2017; Figure 1). Indeed, 1 h of FB1 treatment altered the levels of LCBs in Arabidopsis (Shi et al., 2007). Interestingly, like treatment with FB1, infection with *Pseudomonas syringae* pv. *tomato* DC3000 also provoked a rapid increase in t18:0 (phytosphinganine) levels, implying that LCBs function in pathogen resistance (Peer et al., 2010).

Fumonisin B1 suppresses the activity of all six known mammalian CerSs and therefore, LC and VLC sphingolipid levels decreased in response to FB1 treatment. Moreover, FB1 treatment resulted in the specific inhibition of C16:0 Cer biosynthesis and cured *adipose triglyceride lipase* macrophages from mitochondrial dysfunction and PCD (Aflaki et al., 2012), suggesting that FB1 counters C16 ceramide-mediated cell death. However, the situation is quite different in plants, since FB1 significantly increases C16 ceramide levels in plants. FB1 treatment or depletion of LOH1 and LOH3 resulted in a reduction in VLCF Cer levels (Markham et al., 2011). The levels of Cers and hCer with very long fatty acid chains decreased slightly after 24 h of FB1 treatment (Markham et al., 2011). Significant increases in the levels of 16:0-containing Cer and GluCer were observed in Arabidopsis *loh1* null mutants (Ternes et al., 2011). Furthermore, a stronger depletion of VLC sphingolipids and enhanced accumulation of 16:0 sphingolipids were detected in FB1-treated *loh1-1 loh3-1* double mutants (Markham et al., 2011).

### LCBs Primarily Contribute to the Cytotoxicity of FB1 in Plants

**FIGURE 2:**
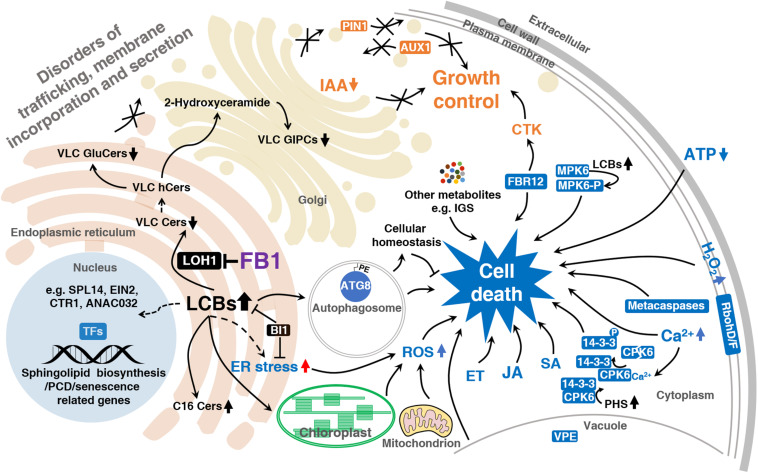
Model summarizing how FB1 impairs and kills plant cells.

Long-chain bases serve as building blocks of sphingolipids and act as second messengers. These compounds are primarily responsible for FB1-induced cell death (Figure 2). The regulation of LCB homeostasis is as essential in plants as it is in animal models. Although the exact molecular mechanism underlying how LCBs control cell fate in plants is far from clear, it is assumed that LCB accumulation would kill plant cells, as it does in animals. The observation that FB1 treatment leads to LCB accumulation and cell death could be tentatively explained by the disequilibrium of the tightly regulated intracellular balance between free LCBs and Cers.

Early insight into the relationship between FB1 and the sphingolipid pathway in plants came from genetic studies focused on identifying Arabidopsis FB1-resistant mutants. *FB1-resistant 11* was characterized as a deletion mutant in the gene encoding a long-chain base1 (LCB1) subunit of SPT. SPT mediates FB1-initiated PCD by catalyzing the first rate-limiting step of *de novo* sphingolipid biosynthesis (Shi et al., 2007; Saucedo-García et al., 2011b; Kimberlin et al., 2013, 2016; Li et al., 2016; Shao et al., 2019; Table 1). For example, increasing SPT activity by overexpressing small subunit of SPT (*ssSPTa*) resulted in the accumulation of LCBs and reduced tolerance to FB1, whereas *ssSPTa* suppression lines displayed lower levels of LCBs but enhanced tolerance to FB1 (Kimberlin et al., 2013). Notably, among all SPTs, only fumonisin b1 resistant 41 (FBR41) represses FB1-elicited cell death when overexpressed, consistent with its negative effect on SPT activity.

**TABLE 1:** Sphingolipid metabolism genes involved in plant responses to FB1.

Gene name	Function	Species	Mutant/transgenic plants	FB1 response	System	References
*LCB1/FBR11*	Subunit of serine palmitoyltransferase	At	*fbr11-1*	Tolerant	Leaves, seedlings	Shi et al., 2007
*LCB2a*	Subunit of serine palmitoyltransferase	At	*lcb2a-1, -2, -3*	Tolerant	Seedlings	Saucedo-García et al., 2011a,b
*LCB2b/*	Subunit of serine	At	dominant mutant fbr41	Tolerant	Leaves, seedlings	Shao et al., 2019
*FBR41*	palmitoyltransferase	At	*FBR41 OE*	Tolerant	Leaves	Shao et al., 2019
*ssSPTa*	Small subunit of serine	At	ssSPTa RNAi	Tolerant	Seedlings	Kimberlin et al., 2013
	palmitoyltransferase	At	ssSPTa-OE	Sensitive	Seedlings	Kimberlin et al., 2013
*ORM1*	Orosomucoid-like protein	At	ORM1-OE	Tolerant	Seedlings	Kimberlin et al., 2016; Li et al., 2016
*ORM2*	Orosomucoid-like protein	At	ORM2-OE	Tolerant	Seedlings	Kimberlin et al., 2016; Li et al., 2016
*ORM1/*	Orosomucoid-like protein	At	*orm1 amiR-ORM2*	Sensitive	Seedlings	Li et al., 2016
*ORM2*			ORM RNAi			
*LCBK1*	Long-chain base kinase	At	LCBK1-KD	Sensitive	Leaves, seedlings	Yanagawa et al., 2017
		At	LCBK1-OE	Tolerant	Leaves, seedlings	Yanagawa et al., 2017
*SPHK1*	Sphingosine kinase 1	At	*SPHK1-KD, sphk1-1*	Tolerant	Leaves	Qin et al., 2017
		At	*SPHK1-KD*	Sensitive	Leaves	Glenz et al., 2019
		At	SPHK1 OE	Sensitive	Leaves	Qin et al., 2017; Glenz et al., 2019
*SPHK2*	Sphingosine kinase 2	At	*sphk2-1*	Tolerant	Leaves	Qin et al., 2017
		At	SPHK2 OE	Sensitive	Leaves	Qin et al., 2017
*SPP1*	Sphingoid phosphate	At	*Spp1*	Tolerant	Leaves	Yanagawa et al., 2017
	phosphatase	At	*SPPASE RNAi (SPHK1-OE)*	Sensitive	Leaves	Qin et al., 2017
		At	*SPP1*-OE	Sensitive	Leaves	Glenz et al., 2019
*DPL1*	Dihydrosphingosine-1-phosphate lyase	At	*dpl 1-1, -2, DPL1 RNAi (SPHK1-OE)*	Sensitive	Leaves	Tsegaye et al., 2007; Nakagawa et al., 2012; Qin et al., 2017; Yanagawa et al., 2017; Glenz et al., 2019
*LOH2*	Ceramide synthase	At	*loh2-1, -2*	Sensitive	Leaves	Mase et al., 2013
		At	LOH2-OE	Tolerant	Seedlings	Luttgeharm et al., 2015
*LOH3*	Ceramide synthase	At	LOH3-OE	Tolerant	Seedlings	Luttgeharm et al., 2015
*ACER*	Alkaline ceramidase	At	amiR-*ACER*-1, *acer-1*	Sensitive	Seedlings, Protoplasts	Wu et al., 2015a
		At	ACER-OX-1	Tolerant	Seedlings, Protoplasts	Wu et al., 2015a
*NCER*	Neutral ceramidase	At	*ncer2*	Sensitive	Seedlings	Zienkiewicz et al., 2019
*BI1*	Protein that interacts with sphingolipid-modifying enzymes	At	*atbi1-l, atbi1-2*	Sensitive	Leaves	Watanabe and Lam, 2006
*PAS1*	*PASTICCINO1*, required for VLCF synthesis	At	*Pas1*	Sensitive	Roots	Markham et al., 2011

*KD, knockdown; OE or OX, overexpressing line. At, *Arabidopsis thaliana*.*

Orosomucoid proteins (ORMs) negatively regulate SPT activity to maintain sphingolipid levels in humans and yeast, and are required for sphingolipid homeostasis in plants, as well as FB1 resistance (Kimberlin et al., 2016; Li et al., 2016). The tolerance of Arabidopsis *ORM* overexpression plants to FB1 was accompanied by reduced levels of C16 Cers, LCBs, and their phosphorylated counterparts (Kimberlin et al., 2016). By contrast, *AtORM* RNA interference lines were more sensitive to FB1 treatment and contained more C16 Cers, LCBs, and LCB-Ps than the wild type (Kimberlin et al., 2016).

In addition to SPT, many enzymes related to LCB metabolism control plant responses to FB1. Overexpressing *AtLCBK1* (*A. thaliana* sphingoid LCB kinase) increased plant resistance to FB1 treatment, whereas *AtLCBK1* knockdown plants showed increased sensitivity to FB1 treatment (Yanagawa et al., 2017). Moreover, analysis of transgenic plants with altered levels of proteins including long-chain base kinase 1 (LCBK1), sphingoid phosphate phosphatase (SPP1), and dihydrosphingosine phosphate lyase (DPL1), which are involved in maintaining LCB/LCB-P homeostasis, revealed a positive correlation between the levels of free LCBs and the degree of FB1-induced cell death (Yanagawa et al., 2017). FB1-induced PCD is primarily due to the accumulation of free LCBs. Directly feeding plants with the free sphingoid bases dihydrosphingosine, phytosphingosine, and sphingosine efficiently induced cell death (Shi et al., 2007). Overexpressing *LCBK1* in Arabidopsis led to hyposensitivity to FB1, whereas knockdown of *LCBK1* conferred hypersensitivity to FB1 (Yanagawa et al., 2017).

Fumonisin B1-elicited cell death is mainly ascribed to the accumulation of dihydroxy-LCBs, but not trihydroxy-LCBs (Saucedo-García et al., 2011b). For instance, the *sbh1-1* mutant, which is deficient in one of the two LCB hydroxylase genes (*SBH1*), contains lower levels of trihydroxy-LCBs and higher levels of total LCBs than wild-type plants (Chen et al., 2008). However, these plants appeared similar to wild-type plants when exposed to FB1 (Saucedo-García et al., 2011b).

Phosphorylated LCB derivatives appear to block cell death induced by the corresponding LCB in a dose-dependent manner, implying that the maintenance of homeostasis between a free sphingoid base and its phosphorylated derivative determines cell fate (Shi et al., 2007). The application of S1P or inhibitors of sphingosine kinase reduced apoptotic-like PCD and promoted cell survival in Arabidopsis cell suspension cultures (Alden et al., 2011). However, the antagonistic effects of phosphorylated LCBs after simultaneous treatment with non-phosphorylated LCBs were due to the reduced uptake of non-phosphorylated LCBs into the tissue (Glenz et al., 2019). Increasing the *in vivo* levels of phosphorylated LCBs did not reduce FB1-induced cell death (Glenz et al., 2019). Analysis of FB1-treated Arabidopsis lines with perturbed levels of phosphorylated LCBs highlighted a positive correlation between non-phosphorylated LCB levels and FB1-elicited cell death, rather than a negative correlation between phosphorylated LCB levels and this process (Glenz et al., 2019).

### FB1 as a Tool to Investigate Sphingolipid Metabolism

Deciphering the role of a putative LCB/Cer metabolic enzyme in FB1 resistance is an excellent first step in confirming its function (Table 1). Ceramidases such as ACER maintain the homeostasis between LCB and Cer. *ACER1*-silenced plants and *acer1* mutant plants were sensitive to FB1, and *ACER1*-overexpressing plants were relatively resistant to FB1 (Wu et al., 2015a). Disrupting neutral ceramidase 2 (NCER2), which likely functions as a ceramide synthase, increased sensitivity to FB1 in Arabidopsis (Zienkiewicz et al., 2019). Bax Inhibitor-1 (BI-1), an evolutionarily conserved cell death suppressor in mammals and plants (Kawai-Yamada et al., 2001; Mosblech et al., 2009; Christensen and Kolomiets, 2011), attenuates cell death progression triggered by FB1 in plants (Watanabe and Lam, 2006). Knockout mutants of *AtBI1* (*atbi1-1* and *atbi1-2*) exhibited increased sensitivity to FB1, whereas these phenotypes were rescued by the overexpression of *AtBI1* (Watanabe and Lam, 2006). Similar to its function in mammals (Chae et al., 2004; Bailly-Maitre et al., 2006), BI-1 helps protect plant cells from ER stress-induced apoptosis (Gao et al., 2007, 2009). The ER membrane-localized protein BI-1 modifies sphingolipids by interacting with FATTY ACID HYDROXYLASE 1 (FAH1) and FAH2 via the electron donor cytochrome b5 (Cb5) in plant cells. BI-1 contributes to sphingolipid biosynthesis during cold stress in Arabidopsis by interacting with ceramide-modifying enzymes (Nagano et al., 2014). Moreover, BI-1 regulates VLCF synthesis by forming a complex with VLCF-synthesizing enzymes (Nagano et al., 2019).

## FB1 Induces Cell Death in Plants

Fumonisin B1 induces cell death in a process involving multiple signaling pathways (Table 2 and Figure 2).

**TABLE 2:** Genes in various signaling pathways involved in plant responses to FB1.

Gene name	Function	Species	Mutant/transgenic plants	FB1 response	System	References
*MPK6*	SA, ET	At	*mpk6*	Tolerant	Seedlings	Saucedo-García et al., 2011b
*ACD2*	SA	At	*acd2-2*	Sensitive	Protoplasts	Asai et al., 2000
*CPR1*	SA	At	*cpr1-1*	Sensitive	Protoplasts	Asai et al., 2000
*CPR6*	SA	At	*cpr6-l*	Sensitive	Protoplasts	Asai et al., 2000
*PAD4*	SA	At	*pad4-l*	Tolerant	Protoplasts	Asai et al., 2000
*ETR1*	ET	At	*etr1-1*	Tolerant	Protoplasts	Asai et al., 2000
		At	*etr1-1*	Tolerant	Leaves	Mase et al., 2013
		At	*etr1-1*	Sensitive	Leaves	Plett et al., 2009
		At	*etr1-1*	Sensitive	Seedlings	Wu et al., 2015b
*EIN2*	ET	At	*ein2*	Sensitive	Leaves	Wu et al., 2015b
*EIN3*	ET	At	*ein3*	Tolerant	Leaves	Mase et al., 2013
*EIN4*	ET	At	*ein4-1*	Tolerant	Leaves	Plett et al., 2009
*ETO1*	ET	At	*eto1-1*	Tolerant	Seedlings	Wu et al., 2015b
*CTR1*	ET	At	*ctr1-1*	Tolerant	Seedlings	Wu et al., 2015b
*ERF4/MACD1*	ET	*Nu*	TRV:NuERF4	Tolerant	Leaves	Mase et al., 2013
		*Nu*	MACD1 OE	Sensitive	Leaves	Mase et al., 2013
*ERF102*	ET	At	*erf 102*,	Tolerant	Leaves	Mase et al., 2013
			*erf 102 (loh2)*	Tolerant	Leaves	Mase et al., 2013
*ERF103*	ET	At	*erf 103 (erf 102)*	Tolerant	Leaves	Mase et al., 2013
*JAR1*	JA	At	*jar1-1*	Tolerant	Protoplasts	Asai et al., 2000
*RGLG3*	JA	At	*rglg3*	Tolerant	Leaves	Zhang et al., 2015
		At	RGLG3OE	Sensitive	Leaves	Zhang et al., 2015
*RGLG4*	JA	At	*rglg4*	Tolerant	Leaves	Zhang et al., 2015
		At	RGLG4OE	Sensitive	Leaves	Zhang et al., 2015
*RGLG3/4*	JA	At	*rglg3 rglg4*	Tolerant	Leaves	Zhang et al., 2015
*COI1*	JA	At	*coi1-2*	Tolerant	Leaves	Zhang et al., 2015
*MYC2*	JA	At	*myc2-2*	Sensitive	Leaves	Zhang et al., 2015
*CPK3*	Ca^2+^	At	*cpk3-1, cpk3-2*	Tolerant	Leaves	Lachaud et al., 2013
*VPE*	Vacuole	At	VPE-null mutant	Tolerant	Leaves	Kuroyanagi et al., 2005
*ATG5*	Autophagy	At	*atg5*	Sensitive	Leaves	Lenz et al., 2011
*ATG10*	Autophagy	At	*atg10*	Sensitive	Leaves	Lenz et al., 2011
*ATG18*	Autophagy	At	*atg18*	Sensitive	Leaves	Coll et al., 2014
			*Atg18-1*	Sensitive	Leaves	Lenz et al., 2011
*ATG18* and *MC1*	Autophagy and Metacaspases		*atg18 mc1*	Sensitive	Leaves	Coll et al., 2014
*MC1*	Metacaspases	At	*Mc1*	Sensitive	Leaves	Coll et al., 2014
*MC4/MCP2d*	Metacaspases	At	*mcp2d-1, mc_*p*_2d-3*	Tolerant	Seedlings	Watanabe and Lam, 2011
		At	AtMCP2d OE	Sensitive	Leaves	Watanabe and Lam, 2011
*KTI1*	Kunitz Trypsin Inhibitor	At	*kti1* RNAi	Sensitive	Leaves	Li et al., 2008
		At	KTI1 OE	Tolerant	Leaves	Li et al., 2008
*RD21*	Papain-like cysteine protease	At	*rd21-1, -4*	Sensitive	Root	Ormancey et al., 2019
*LSD1*	Zinc finger protein	*Os*	*OsLSD1*	Tolerant	Tobacco Seedlings	Wang et al., 2005
*SPL14/FBR6*	Transcription	At	*fbr6*	Tolerant	Seedlings	Stone et al., 2005
ANAC032	Transcription, senescence	*At*	ANAC032 SRDX lines	Tolerant	Seedlings	Mahmood et al., 2016
*CYP79B2/CYP79B3*	Glucosinolates	At	*cyp79B2 cyp79B3*	Sensitive	Leaves	Zhao et al., 2015
*TGG1 TGG2*	Glucosinolates	At	*tgg1 tgg2*	Sensitive	Leaves	Zhao et al., 2015
*UGP1*	UDP-glucose pyrophosphorylase	At	*SALK_020808 SALK_100183*	Tolerant	Leaves	Chivasa et al., 2013
ATP SYNTHASE β- SUBUNIT	ATP synthase	At	*SALK_024990*	Tolerant	Leaves	Chivasa et al., 2011
			*SALK_135351 SALK_005252*			
*CYCLASE1*	Cyclase-family protein, SA, ATP	At	*cyclase1-1, -2*	Sensitive	Leaves	Smith et al., 2015
*RING1*	RING motif protein	At	amiR-R1^159^ #1-16, #39-1	Tolerant	Seedlings	Lin et al., 2008
		At	RING1-3HA #2-26, #4-1	Sensitive	Seedlings	Lin et al., 2008
*DAL1*	Ring finger protein		*dal1-1, -2*	Sensitive	Leaves	Basnayake et al., 2011
*DAL2*	Ring finger protein		*dal2-1, -2*	Sensitive	Leaves	Basnayake et al., 2011
*BI1*	ER stress	At	*atbi1-1*/*atbi1-2*	Sensitive	Leaves	Watanabe and Lam, 2006; Nagano et al., 2009
*RabG3b*	GTPase, ATG	At	constitutively active RabG3b (RabG3bCA) and RabG3b OE *sad2-2*, *-5*	Sensitive	Leaves	Kwon et al., 2009; Kwon et al., 2013
*SAD2*	Importin beta-like protein	At		Sensitive	Seedlings	Zheng et al., 2019
*ILP*	Inhibitor of apoptosis-like protein, ring finger protein	At	*ILP*	Tolerant	Seedlings	Kim et al., 2011
*ATR1*	Unknown	At	*Atr1*	Tolerant	Seedlings	Gechev et al., 2008

*KD, knockdown; OE or OX, overexpressing line. At, *Arabidopsis thaliana*.*

### Hormone Signaling

The link between ethylene and plant responses to FB1 is well known. Initially, it was reported that ethylene-dependent signaling pathways are required for FB1-induced cell death in Arabidopsis protoplasts (Asai et al., 2000). Arabidopsis ethylene receptors play distinct roles in FB1-induced cell death (Plett et al., 2009). Intriguingly, different reports describe different phenotypes of *ethylene response 1-1* (*etr1-1*) mutants, possibly due to different growth conditions such as photoperiod (Asai et al., 2000; Plett et al., 2009; Mase et al., 2013; Wu et al., 2015b). By investigating the responses of various ethylene mutants to FB1 and the corresponding sphingolipid profiles of the plants, as well as the effects of 1-aminocyclopropane-1-carboxylic acid on FB1-induced cell death and sphingolipid metabolism, our group demonstrated that ethylene signaling inhibits sphingolipid synthesis, thereby playing a negative role in FB1-induced cell death (Wu et al., 2015b). Despite the finding that ethylene signaling modulates sphingolipid metabolism, the possibility that ethylene signaling acts downstream of sphingolipids (such LCBs and Cers) cannot be excluded. Notably, C24:1-Cer interacts with constitutive triple response 1 (CTR1) and inhibits its kinase activity, thereby modulating CTR1-mediated ethylene signaling (Xie et al., 2015), supporting this hypothesis.

The role of the plant hormone jasmonate (JA) in plant resistance to fungi is well known (Pieterse et al., 2009). The role of JA in mediating FB1-induced PCD was first described by Asai et al. (2000). The authors found that Arabidopsis protoplasts isolated from jasmonate resistant 1-1 (*jar1-1*) plants, which are insensitive to JA, exhibited much lower susceptibility to cell death induced by FB1 than FB1-treated wild-type protoplasts (Asai et al., 2000). Similarly, the deletion of oxophytodienoate reductase 3 (*OPR3*), which is involved in JA biosynthesis, enhanced the resistance of protoplasts to FB1 upon a dark/light shift (Danon et al., 2004). However, these findings may need to be further explored, since OPR3 is only partially required for JA biosynthesis and data obtained with the *opr3* mutants are often unclear (Chini et al., 2018). FB1 hijacks the JA pathway to initiate PCD. FB1 induces JA-responsive defense genes but represses growth-related and JA biosynthesis-related genes, thereby reducing JA contents in plants (Zhang et al., 2015). Furthermore, the ubiquitin ligases ring domain ligase 3 (RGLG3) and RGLG4 coordinately and positively regulate FB1-triggered PCD by modulating the JA signaling pathway in a coronatine insensitive 1 (COI1)- and MYC2-dependent manner in Arabidopsis (Zhang et al., 2015).

In addition to ethylene and JA, plant susceptibility to FB1 appears to be associated with salicylic acid (SA) signaling, whereas FB1-induced PCD does not require the SA signal transmitter non-expresser of PR genes 1 (Asai et al., 2000). Indeed, SA promotes FB1-induced cell death (Smith et al., 2015). Moreover, FB1-induced SA likely suppresses the JA pathway to facilitate cell death, since exogenously applied SA inhibited JA signaling additively with FB1 treatment (Zhang et al., 2015).

Taken together, these findings indicate that FB1-elicited cell death requires ethylene, JA, and SA. Nevertheless, the molecular mechanisms underlying the roles of these hormone-signaling pathways in FB1-induced cell death remain to be investigated.

### MPK6, Ca^2+^, Reactive Oxygen Species, and ATP Signaling

Mitogen-activated protein kinases (MAPKs) were activated in FB1-treated monkey kidney samples, but the functional role of MAPKs in FB1-induced toxicity has not been addressed in this system (Yin et al., 2016). In Arabidopsis, MAP kinase 6 (MPK6) was described as a novel transducer in the pathway leading to LCB-induced PCD (Saucedo-García et al., 2011b). In addition to mediating PCD downstream of LCBs, MPK6 can be activated by phytosphingosine−1−phosphate (PHS−P), a response rapidly and transiently evoked by chilling (Dutilleul et al., 2012). Nevertheless, how MPK6 triggers FB1-induced cell death downstream of PCD signaling remains unknown.

Calcium signaling is also required for FB1-induced cell death. Upon exposure to FB1, intracellular Ca^2+^ levels increase via FB1-induced phytosphingosine (PHS) (Lachaud et al., 2013). Meanwhile, PHS activates calcium-dependent kinase 3 (CPK3), which phosphorylates its associated partners, the 14-3-3 proteins, thus leading to the disruption of the 14-3-3-CPK3 complex and CPK3 degradation, ultimately triggering PCD in plants (Lachaud et al., 2013).

Reactive oxygen species (ROS) have key roles in PCD; in fact, cell death is considered to be triggered by ROS (Mittler, 2017) and ROS production precedes LCB-induced PCD in many cases (Shi et al., 2007; Saucedo-García et al., 2011a). The cytosolic increase in Ca^2+^ levels and the accumulation of ROS are critical for inducing PCD. The disruption of Arabidopsis super sensitive to aba and drought 2 (*SAD2*) (an importin beta-like gene) enhanced H_2_O_2_ accumulation and cell death in the *sad2-5* mutant under FB1 treatment (Zheng et al., 2019). The FB1 sensitivity of *sad2-5* is partially dependent on Ca^2+^ signaling (Zheng et al., 2019). SAD2-mediated Ca^2+^ and ROS signaling appear to function downstream of LCB, since the *sad2-5 fbr11-1* double mutant exhibited the same FB1-insensitive phenotype as *fbr11-1* (Zheng et al., 2019). In fact, many signaling pathways involved in regulating FB1-induced cell death are associated with ROS. For instance, the breakdown products of indole glucosinolate (IGS), which function in innate immune responses (Clay et al., 2009), attenuate FB1-induced cell death through their ROS-scavenging activity and function independently of indole-3-acetic acid signaling (Zhao et al., 2015).

Fumonisin B1 triggers the rapid depletion of extracellular ATP in Arabidopsis, which initiates cell death; this process was rescued by exogenous ATP treatment (Chivasa et al., 2005). ADP, AMP, and inorganic phosphate had no effect on this process, indicating that intact ATP is required for the rescue of FB1-induced cell death. In line with this notion, Arabidopsis knockout mutants lacking the gene encoding the mitochondrial ATP synthase β-subunit are resistant to FB1 (Chivasa et al., 2011). ATP and SA antagonistically regulate the cell death process (Smith et al., 2015). Further study based on this hypothesis revealed that CYCLASE1, an extracellular matrix proteins whose response to SA is suppressed by ATP, is a negative regulator of FB1-induced cell death (Smith et al., 2015).

### Autophagy, Disruption of the Vacuolar Membrane, and ER Stress

Autophagy, a mechanism used to degrade unwanted constituents in eukaryotic cells, is essential for maintaining cell homeostasis and nutrient recycling. We recently demonstrated that FB1 and LCBs induce autophagy in Arabidopsis (Zheng et al., 2018). The loss of Arabidopsis ACER, an ACER that hydrolyzes Cer to LCB, inhibited autophagy, and its overexpression promoted autophagy under various abiotic stress conditions (Zheng et al., 2018).

In animals, the induction of autophagy has either cytoprotective or cytotoxic effects in certain types of mycotoxin-mediated cytotoxicity, but how the role (pro-survival or pro-death) of autophagy in regulating cell death is determined remains elusive (Yin et al., 2018). In plants, autophagy has also been assigned pro-death and pro-survival functions in controlling cell death (Zeng et al., 2019). Treatment with FB1 caused spreading lesions to form in several *autophagy-related* (*atg*) mutants (Lenz et al., 2011; Coll et al., 2014), implying that autophagy constitutes a pro-survival mechanism that contains unrestricted cell death in the presence of FB1. Consistent with this finding, autophagy participates in controlling plant lipid metabolism and catabolism (Have et al., 2019). Cer and GIPC levels are significantly altered in *atg5* mutants in a SA-independent manner (Have et al., 2019). This observation suggests that autophagy modifies the composition of endomembrane lipids, especially plasma membrane lipids, thereby altering the outcome of FB1 treatment. Nevertheless, overexpressing or constitutively active Rab GTPase RabG3b, which contributes to cell death during the hypersensitive response (HR) in Arabidopsis by activating autophagy, led to unrestricted hypersensitive PCD in response to FB1 (Kwon et al., 2009, 2013), confirming another role of autophagy in FB1-associated cell death. In summary, autophagy functions in life and death of the cell through multiple pathways, but the underlying molecular mechanisms remain to be revealed.

The disruption of vacuolar membranes is one of the early events in FB1-induced cell death, followed by lesion formation (Kuroyanagi et al., 2005). The deletion of all four vacuolar processing enzyme (VPE) genes or treatment with VPE-specific inhibitors significantly suppressed FB1-induced cell death in Arabidopsis (Kuroyanagi et al., 2005). VPE exhibits caspase-1 activity and is required for the collapse of the tonoplast during FB1 treatment (Kuroyanagi et al., 2005). Treatment with caspase-1 inhibitors prevented the formation of FB1-induced lesions, indicating that caspase activity is required during FB1-induced cell death (Kuroyanagi et al., 2005). Notably, a deficiency of the retromer complex leads to defects in late endocytic/lytic compartments and impairs autophagy-associated vacuolar processes (Munch et al., 2015). vacuolar protein sorting 35, part of the retromer complex, functions in endosomal protein sorting and vacuolar trafficking and is involved in *acd11*-associated cell death (Munch et al., 2015). It would be interesting to investigate whether this process is required for FB1 resistance.

In animals, ER stress is responsible for autophagy and autophagic cell death induced by FB1 (Yin et al., 2016). Although we lack direct evidence in plants, the accumulation of LCB appears to increase ER stress-related responses. We frequently observed irregular ER and vacuolization in *orm1* amiR-ORM2 cells, with many tiny membrane sacs located close to the plasma membrane and cell wall (Li et al., 2016). In line with this observation, the expression of ER stress marker genes significantly increased in *orm1* amiR-ORM2 plants compared to the wild type (Li et al., 2016). Apart from mediating sphingolipid metabolism, AtBI1 plays a pivotal role as a highly conserved survival factor during ER stress that acts in parallel with the unfolded protein response pathway. Compared to wild-type plants, *atbi1-1* and *atbi1-2* exhibit hypersensitivity to tunicamycin, an inducer of ER stress, whereas overexpressing *AtBI1* markedly reduced the sensitivity of Arabidopsis seedlings to tunicamycin (Watanabe and Lam, 2008). Whether the role of BI1 in the ER stress response is associated with its sphingolipid-modifying function remains to be investigated.

### Other Participants in FB1-Induced PCD

Plant proteases, such as serine proteases and cysteine proteases, have been implicated in PCD (Rotari et al., 2005). The serine protease kunitz trypsin inhibitor 1 (KTI1) acts as a functional protease in Arabidopsis when produced in bacteria and *in planta*. RNAi silencing of *KTI1* resulted in enhanced lesion development in plants after FB1 treatment (Li et al., 2008). Conversely, overexpressing *AtKTI1* reduced lesion development in plants after FB1 treatment (Li et al., 2008). The cysteine protease metacaspase 2D (MCP2d)/MC4 also participates in FB1-induced PCD. The AtMCP2d mutants *mcp2d-1* and *mcp2d-3* show reduced sensitivity to FB1, whereas *AtMCP2d* overexpression lines are more sensitive to FB1 and display accelerated progression of cell death (Li et al., 2008). AtMCP2d exclusively localizes to the cytosol (Li et al., 2008). AtMCP2d is engaged in both oxidative stress-induced cell death and pathogen-induced cell death (Li et al., 2008). The type I metacaspase AtMC1 likely plays a positive role in the HR, methyl viologen-induced cell death, and FB1-induced cell death (Coll et al., 2014). Finally, the Arabidopsis papain-like cysteine protease responsive to desiccation 21 is a negative regulator of FB1-induced cell death (Ormancey et al., 2019). Together, these findings support the notion that the cell death triggered by FB1 requires general cell death factors, such as various proteases.

Membrane microdomains have also been implicated in PCD. RING1, a RING finger domain protein with E3 ligase activity, is localized to the lipid rafts of plasma membranes (Lin et al., 2008). Knockdown of *RING1* led to FB1 hyposensitivity, whereas overexpressing *RING1* conferred hypersensitivity to this treatment (Lin et al., 2008). RING1 acts as a signal from lipid rafts in the plasma membrane that trigger the FB1-induced plant PCD pathway (Lin et al., 2008). Transient expression of *Capsicum annuum RING1* induced cell death in pepper leaves (Lee et al., 2011). It would be interesting to further investigate how lipid rafts in the plasma membrane respond to FB1. Suppressor of PPI1 locus 1 (SP1) regulates chloroplast biosynthesis, and both SP1 and SP1-like 1 (SPL1) regulate peroxisome biosynthesis in Arabidopsis (Ling et al., 2012, 2019; Pan et al., 2016, 2018). These proteins negatively regulate FB1-induced PCD as well (Basnayake et al., 2011). The involvement of these two regulators of organelle biogenesis in FB1-induced cell death points to a possible link between organelles and FB1 resistance.

Various proteins involved in FB1-induced cell death, such FBR6 and FBR12, also regulate plant growth and development. Misexpression of *AtSPL14*, encoding a plant-specific SQUAMOSA promoter (SBP)-domain putative transcription factor, conferred FB1 resistance in *fbr6* plants (Stone et al., 2005). In addition to participating in FB1-associated cell death, AtSPL14 modifies normal plant development, as the *fbr6* mutant displays altered plant architecture in the absence of FB1, most notably elongated petioles and enhanced leaf margin serration (Stone et al., 2005). These findings point to a possible association between insensitivity to FB1-induced cell death and altered plant development. Similar observations were made for Arabidopsis *fbr12*, a mutant deficient in eukaryotic translation initiation factor 5A. FBR12 positively regulates PCD caused by infection with virulent *P. syringae* pv. *tomato* DC3000 (*Pst* DC3000), and transgenic plants constitutively overexpressing *FBR12* exhibited phenotypes consistent with precocious cell death (Hopkins et al., 2008).

FBR12 mediates processes beyond cell death, such as cell division and cell growth (Feng et al., 2007). The *fbr12* mutant is extremely dwarf, with substantially reduced sizes and numbers of all adult organs, and shows abnormal floral organ development and defective sporogenesis, leading to the abortion of both female and male germline cells during reproductive development (Feng et al., 2007). FBR12 also functions in cytokinin-mediated specification of the root protoxylem, as a mutation in *FBR12* led to defective protoxylem development and reduced sensitivity to cytokinin (CTK) (Ren et al., 2013). These findings point to a connection between PCD and inhibited development induced by FB1.

The solid arrows indicate established links and the dashed arrows indicate putative links. Reduced VLC sphingolipid levels lead to disordered trafficking, membrane incorporation, and secretion. LCBs produced in response to FB1 to trigger cell death signaling, such as ET, JA, SA, ROS, Ca^2+^, ATP signaling, and so on. LCBs regulate the activities of cell death-associated proteins such as MPK6, CPK6, and 14-3-3 proteins. LCBs also induce autophagosome formation, which maintains cellular homeostasis, protecting the cell from unnecessary death. ER stress appears to be involved in this process. LCBs induce the expression of sphingolipid biosynthesis, PCD, and senescence-related genes through transcription factors such as EIN2, CTR1, SPL14, and ANAC032. 14-3-3, 14-3-3 family protein; ANAC032, NAC domain containing protein 32; ATG8, autophagy 8; AUX1, auxin resistant 1; BI1, bax inhibitor 1; Cers, ceramides; CPK6, calcium dependent protein kinase 6; CTK, cytokinin; CTR1, constitutive triple response 1; EIN2, ethylene insensitive 2; ET, ethylene, ER stress, endoplasmic reticulum stress; FB1, fumonisin B1; FBR12, fumonisin B1 resistant 12; GluCers, glucosylceramides; GIPCs, glycosyl-inositolphosphoryl-ceramides; hCers, hydroxyceramides; IAA, indole-3-acetic acid; IGS, indole glucosinolate; JA, jasmonate; LCBs, long-chain bases; LOH1, LAG1 Homolog 1; MPK6, MAP kinase 6; MPK6-P, MPK6 phosphorylated form; PE, phosphatidylethanolamine; PHS, phytosphingosine; PIN1, PIN-FORMED 1; RbohD/F, respiratory burst oxidase homolog; ROS, reactive oxygen species; SA, salicylic acid; SPL14, squamosa promoter binding protein-like 14; TFs, transcription factors; VLC, very-long-chain; VPE, vacuolar processing enzyme.

## FB1 Impairs Plant Growth and Affects Plant Development

Sphingolipids, particularly those containing VLCFAs, are essential for protein sorting and secretion in eukaryotes. The hydrophobicity, membrane-leaflet interdigitation, and transition of a membrane from a fluid to a gel phase, which are required for microdomain formation, are highly correlated with the presence of VLC sphingolipids (Markham et al., 2011). Several reports speculated that sphingolipids may represent the main reservoir of VLCFAs in leaves; since phosphatidylserine and phosphatidylethanolamine harbor VLCFAs as well in their backbone and no quantitative comparison with these lipid classes exist, this needs to be analyzed in more detail in the future (Pata et al., 2010; Maatta et al., 2012; Cacas et al., 2016; De Bigault Du Granrut and Cacas, 2016). Plants overexpressing *LOH1* and *LOH3* showed increased growth and cell division, with enhanced production of Cers with VLCFAs and trihydroxy LCBs (Luttgeharm et al., 2015). Interestingly, unlike *LOH2*, overexpressing *LOH1/3* had only a minor effect on the sphingolipid profiles of plants.

Fumonisin B1-mediated reductions in VLCFA levels remodel the membrane structure and influence numerous processes that require the foundation maintained by VLC sphingolipids (Figure 2). Upon FB1 application, tobacco (*Nicotiana tabacum*) BY2 cells showed severe effects on cell growth and cell shape and a delay in cell division (Aubert et al., 2011). These changes were accompanied by the formation of ER-derived tubular aggregates, as well as inhibited ER-to-Golgi cargo transport (Aubert et al., 2011). A change in the polar localization of the auxin transporter PIN-FORMED 1 (PIN1) was also observed, but there was little effect on endocytic processes. FB1 targets molecules distinct from those targeted by Brefeldin A, an ER-to-Golgi transport inhibitor (Ritzenthaler et al., 2002; Langhans and Robinson, 2007), as revealed by electron microscopy. These findings reflect the importance of sphingolipids in cell growth and the establishment of cell polarity in plant cells, especially their contribution to the functional organization of the ER or its differentiation into distinct compartments (Aubert et al., 2011).

The reduction in VLC sphingolipid levels leads to auxin-dependent inhibition of lateral root emergence, which is correlated with the selective aggregation of the plasma membrane auxin carriers auxin resistant 1 (AUX1) and PIN1 in the cytosol (Markham et al., 2011). Defective targeting of polar auxin carriers is characterized by the specific aggregation of Rab-A2a- and Rab-A1e-labeled early endosomes along the secretory pathway (Markham et al., 2011). The formation of these aggregates is associated with the accumulation of membrane structures and vesicle fragmentation in the cytosol (Markham et al., 2011), indicating that sphingolipids with very long acyl chains define a trafficking pathway with specific endomembrane compartments and polar auxin transport protein cargoes (Markham et al., 2011). Finally, VLC sphingolipids, particularly VLC glycosphingolipids, are essential structural determinants of vesicle dynamics and membrane fusion during cytokinesis (Molino et al., 2014). Inhibiting VLC sphingolipids biosynthesis by FB1 led to the formation of defective cell plates with persistent vesicular structures and large gaps (Molino et al., 2014).

Route 1 (Orange): FB1-induced decreases in VLCF levels strongly affect membrane properties, which are responsible for normal plant growth. Route 2 (Blue): FB1-induced increases in LCB and C16 Cer levels primarily affect hormone, Ca^2+^, ROS, and ATP signaling, which ultimately contribute to cell death. However, this process is sufficiently complex such that interplay with other factors in each route and even across routes determines the final outcomes. Black, targeted by FB1 (purple), orange, inhibited or reduced by FB1; blue, promoted or elevated by FB1. CerS, ceramide synthase; ET, ethylene, FB1, fumonisin B1; IGS, indole glucosinolate; JA, jasmonate; LCBs, long-chain bases; ROS, reactive oxygen species; SA, salicylic acid; SLs, sphingolipids; VLCFA, very-long-chain fatty acid.

## Maize–*F. verticillioides* Interaction

Fumonisin-producing pathogens are the major causal agents of fusarium ear rot, one of the most important diseases affecting maize production worldwide (Picot et al., 2010). FB1 is the predominant fumonisin in maize kernels (Bartók et al., 2006). The fungal genes required for fumonisin biosynthesis are organized in a cluster designated the fumonisin (FUM) gene cluster, which contains 22 genes with a length of 42 kb (Proctor et al., 1999,2003). This cluster may be regulated by multiple environmental factors including pH, water availability or nutrient sources, and various fungal genes to favor or restrain fumonisin biosynthesis (Picot et al., 2010). Among the 22 genes, 15 are co-regulated, including the key gene *FUM1*, which encodes a polyketide synthase (PKS) (Proctor et al., 1999).

In the complex interaction between *F. verticillioides* and maize, investigation of the role of fumonisin production on disease development has yielded controversial results. Only strains of *F. verticillioides* that produce fumonisins cause foliar disease symptoms on seedlings of the sweet maize hybrid “Silver Queen” (Williams et al., 2006; Lanubile et al., 2012a,b). However, the *FUM1* deletion mutants, which do not produce fumonisin, still cause ear rot, implying that production of fumonisins is not required for ear rot (Jardine and Leslie, 1999; Desjardins et al., 2002). Lanubile et al. (2013) found that *PKS* is a relevant gene, essential not only for the fumonisin biosynthetic pathway, but also for pathogen colonization (Lanubile et al., 2013).

*Sarocladium zeae*, a fungal endophyte of maize, can co-inhabit a seed with *F. verticillioides* due to its capability to produce pyrrocidines A and B, which inhibit the growth of *F. verticillioides* (Gao et al., 2020). *FvZBD1* (*FVEG_00314*) encodes a genetic repressor of fumonisin production, is directly adjacent to the *FUM* cluster, and was induced in response to pyrrocidines. This suggests that other microbes may manipulate the fumonisin biosynthetic program of *F. verticillioides* to control *F. verticillioides* growth in the host (Gao et al., 2020). Interestingly, a high dose of fumonisin induced necrosis and wilting in maize seedlings, but a low dose activated detoxification processes, indicating that maize has recovery mechanisms (Arias et al., 2012).

In maize, batteries of defense genes involved in pathogen-associated molecular pattern-triggered immunity and effector-triggered immunity, as well as multiple signaling, such as MAPK, Ca^2+^, ROS, hormones (including SA, auxin, abscisic acid, ethylene, JA) signaling are activated in response to *F. verticillioides* infection (Lanubile et al., 2017). A complex network of metabolic pathways is required for resistance to *F. verticillioides* (Lanubile et al., 2017). *Lipoxygenase* (*LOX*) genes encode non-heme iron-containing dioxygenases that catalyze the oxygenation of polyunsaturated fatty acids (Vick and Zimmerman, 1983), which are processed into an estimated 400 metabolites including JA (Mosblech et al., 2009). In plants, LOXs are involved in host susceptibility to fungal invasion and mycotoxin production (Kock et al., 2003; Christensen and Kolomiets, 2011; Maschietto et al., 2015). Depending on which carbon on the fatty acid chain is oxygenated, LOXs are classified into two main functional groups, 9-LOXs and 13-LOXs. It seems that a specific plant 9-LOX isoform is required for fungal pathogenesis, including disease development and production of spores and mycotoxins (Gao et al., 2007, 2009; Christensen et al., 2014). In addition, many factors contribute to resistance to *F. verticillioides* and fumonisin contamination, including the biochemical composition of the endosperm, and fatty acid composition, as well as many other metabolites, such phenylpropanoid pathway-related metabolites (flavonoids, phenolic compounds, and phytoalexins), flavones, and 2-amino-3H-phenoxazin-3-one (Lanubile et al., 2017).

## Concluding Remarks and Outlook

Inhibition of ceramide synthesis by FB1 disturbs the homeostasis of sphingolipid metabolism, resulting in changes in structural and signaling sphingolipids, including elevated LCB and LCF Cer levels and decreased formation of Cers and complex VLC sphingolipids, accounting for the broad spectrum of plant disease symptoms. LCBs, as well as LCF Cers, contribute to cell death by functioning as signals, while Cers and complex VLC sphingolipids are critical components of the plant plasma membrane and endomembrane system that are closely associated with the fluidity and biophysical order of the membrane. Thus, the perturbations caused by FB1 not only induce cell death signals, but they also damage the structural components of the cell and impair plant growth. Small molecules and metabolites such as certain hormones (ethylene, JA, SA), ROS, NO, ATP, and Ca^2+^ likely affect FB1-triggered PCD, highlighting the strong association between sphingolipids and cell death. Nevertheless, the molecular details of the underlying mechanism are largely unclear.

In addition to being used as a ceramide inhibitor or a fungal toxicant, FB1 represents a highly useful tool for revealing details about sphingolipid-controlled cell death and growth inhibition. These details would benefit agriculture by helping us overcome the subtle strategy of FB1-producing pathogens and others foes sharing similar mechanisms in plant–pathogen interactions.

**FIGURE 3:**
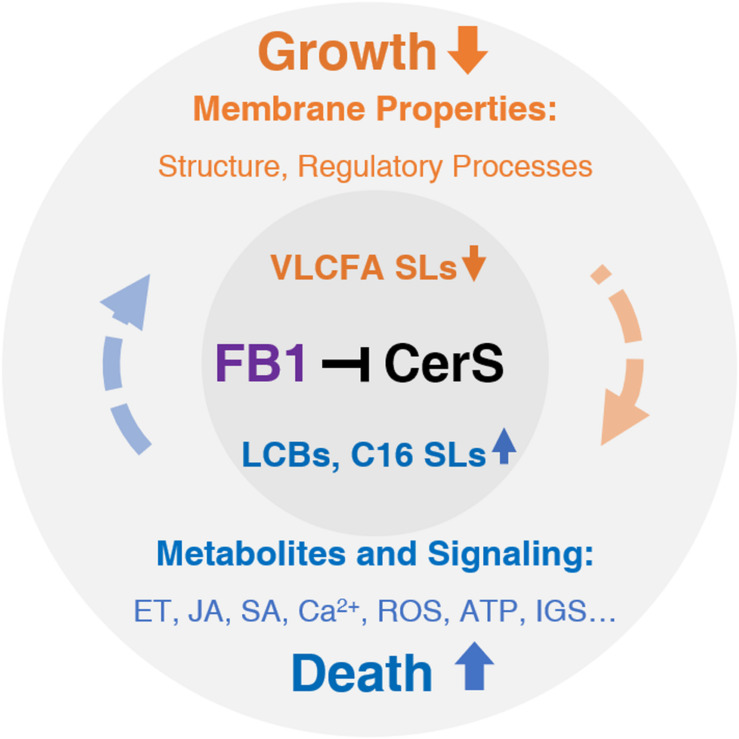
The effects of FB1 treatment.

Based on the complicated signaling network induced by FB1, we propose a model to classify the global effects of this compound into basic two routes (Figure 3). However, it should be noted that most of these events interact with each other in a complex manner. For example, FB1 manipulates the JA signaling pathway through SA signaling (Zhang et al., 2015). In general, the activation of cell death signaling represses plant growth, which in turn sometimes leads to cell death.

Sphingolipids remain an underexplored, large group of metabolites (Ali et al., 2018). The study of FB1, the best-characterized inhibitor of sphingolipid biosynthesis, provides a fascinating illustration of the remarkably sophisticated sphingolipid-signaling network that is established in plants and animals. However, our understanding of the mechanisms underlying FB1 toxicity in plants is still in its infancy in terms of identifying the direct target(s) of FB1 and key elements in the responses to FB1.

Recent studies using molecular genetics and biochemical approaches have enhanced our understanding of this issue in plants and provided a deeper understanding of the roles of sphingolipids in plant life and death. Despite these important findings, our understanding of the importance of sphingolipids for the regulation of plant cell death and growth, and consequently crop yields remains limited and sphingolipids represent an exciting area for further study. Many efforts have been made to functionally characterize individual genes downstream of FB1. However, little is known about the FB1 signal transduction network. Therefore, apart from screening more participants involved in the FB1 response, studies should be designed to elaborate how the known participants operate simultaneously in a coordinated signaling network under FB1 treatment. In particular, it is crucial to investigate the connection between the regulators of FB1 resistance and sphingolipid metabolism. Specifically, sphingolipid profile analysis of FB1-resistant or -sensitive mutants with and without FB1 treatment should be performed to determine whether these proteins engage in sphingolipid metabolism. Furthermore, our lack of knowledge about the molecular targets of individual sphingolipid components, as well as their biological significance to plants, is still a major impediment to understanding FB1 signaling. Thus, there is a need for interactome association analysis between proteins downstream of FB1 and LCB, as well as sphingolipid enzymes, to untangle the interplay between these signals and sphingolipid metabolites or metabolism pathways. Recent studies have demonstrated the great potential of using the photoactivatable and clickable analog of sphingosine (pacSph) and azide-tagged sphingolipids to identify sphingolipid targets (Haberkant et al., 2016; Schulte-Zweckel et al., 2018). In addition to high-throughput screening of sphingolipid-interacting proteins, it will be important to gain deep insight into the roles of essential components of the HR, particularly enhanced disease susceptibility 1, activated disease resistance 1, and non-race-specific disease resistance 1, in FB1-triggered cell death, given that these HR signaling pathways are active in response to FB1.

Beside Arabidopsis, the FB1 signaling network should be elaborated in crop plants. Relatively little is known about the physiological effects of fumonisins from fungal pathogens in the natural environment on the development of plant diseases. The introduction and manipulation of parts of the FB1 signaling pathway in crop plants will have broad and overarching impacts on agriculture to help overcome the effects of FB1-producing pathogens. In view of the broad host range of FB1-producing pathogens, the eradication of plant diseases (as well as animal diseases) caused by FB1 appears to be elusive. However, better insight into the global effects of FB1 on plants will undoubtedly contribute to the development of a rational, effective approach to reducing the hazards of FB1 contamination.

## Author Contributions

H-YZ and NY designed the manuscript and wrote the manuscript. C-YL and NY contributed to the reagents and materials. All authors have discussed and approved the final manuscript.

## Conflict of Interest

The authors declare that the research was conducted in the absence of any commercial or financial relationships that could be construed as a potential conflict of interest.
