# Supplementary material for: Fumonisin B1: A Tool for Exploring the Multiple Functions of Sphingolipids in Plants
Source: Front Plant Sci. 2020 Oct 27;11:600458. doi: 10.3389/fpls.2020.600458 (PMC7652989; doi:10.3389/fpls.2020.600458)
Supplement: Supplementary file 1 [file Image_1.pdf]

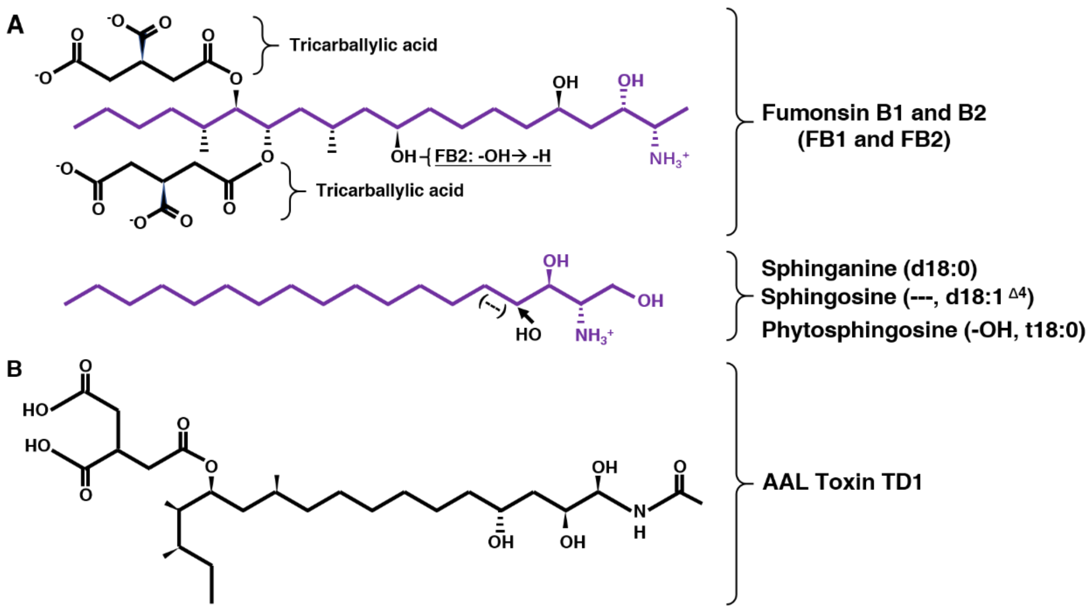

Supplemental Figure S1. Structures of major fumonisins, representative LCBs and AAL toxin.

A. Comparison of the structures of FBs with sphinganine, sphingosine and phytosphingosine. The fumonisin B1 (FB1), FB2, and representative long-chain bases (LCBs) are shown with highlighting of the sphingoid base-like portion in purple. B. Structures of *Alternaria alternata* f.sp. *lycopersici* (AAL) toxin TD1.
